# Intraspecific transitioning of ecological strategies in *Pinus massoniana* trees across restoration stages

**DOI:** 10.1002/ece3.11305

**Published:** 2024-05-05

**Authors:** Sihang Lu, Jiazheng Wang, Ao Liu, Feiya Lei, Rong Liu, Shouzhong Li

**Affiliations:** ^1^ Institute of Geography Fujian Normal University Fuzhou China; ^2^ School of Geographical Sciences Fujian Normal University Fuzhou China; ^3^ State Key Laboratory of Grassland Agro‐ecosystems, College of Ecology Lanzhou University Lanzhou China; ^4^ Yuzhong Mountain Ecosystems Observation and Research Station Lanzhou University Lanzhou China; ^5^ Systems Ecology, Department of Ecological Science VU University Amsterdam The Netherlands

**Keywords:** CSR theory, ecological restoration, functional traits, ontogenetic stages, pine tree, *Pinus massoniana*

## Abstract

Intraspecific variation in plant functional traits and ecological strategies is typically overlooked in most studies despite its pivotal role at the local scales and along short environmental gradients. While CSR theory has been used to classify ecological strategies (competitive C; stress‐tolerant, S; ruderal, R) in different plant species, its ability to explain intraspecific variation in ecological strategies remains uncertain. Here, we sought to investigate intraspecific variation in ecological strategies for *Pinus massoniana*, a pioneer conifer tree for ecological restoration in Changting County, southeast China. By measuring key leaf traits and canopy height of 252 individuals at different ontogenetic stages from three plots spanning distinctive stages along early ecological restoration and calculating their C, S, and R scores, we constructed an intraspecific CSR system. All individual strategies shifted across three restoration stages, with adults from higher S component to higher C component while juveniles from higher S component to higher R component. Our results suggest that while strategies of all *P. massoniana* individuals start with tolerance to environmental stress, as restoration proceeds, adult transition towards completion for light, whereas juveniles shift to an acquisitive resource use. The study reveals an intraspecific pattern of strategy variation during forest restoration, contributing to our understanding of how plants adapt to diverse environments.

## Graphical abstract

CSR theory and its simple quantitative methodology can effectively be used to study the intraspecific variation of strategies in a conifer tree, *Pinus massoniana*. This intraspecific classification of plant strategies provides useful information about how individual plant at different ontogenetic stages adapting to changing environmental conditions. *P. massoniana* trees' strategy is quite plastic phenotypically, as it undergoes shifts in response to stages of ecological restoration. Specifically, adults shift from tolerating environmental stress to competing for light, while juveniles shift from tolerating environmental stress to accumulating biomass through rapidly acquisitive resource use.

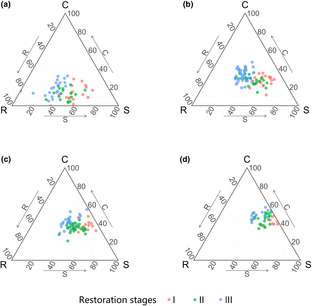

## 1 INTRODUCTION

A plant functional trait is any morphological, physiological, or phenological feature that indirectly affects their fitness through its impact on growth, reproduction, or survival (Violle et al., 2007). Plant ecological strategies can be inferred and conveyed by differing sets of viable trait combinations (Díaz et al., 2016; Grime & Pierce, 2012; Lambers & Poorter, 1992). Most studies of plant traits have focused on interspecific patterns, leaving trait variation within species less known (Hulshof & Swenson, 2010; Kichenin et al., 2013). Recent work has shown that, for the same species, the intraspecific responses of plant traits to environmental changes do not always follow the interspecific pattern (Anderegg et al., 2018). Thus, by ignoring intraspecific trait variation, we may underestimate the ability of species to adjust their ecological strategies via phenotypic responses to local environmental variation, which could lead to misinterpretations of the mechanisms underlying species distributions (Siefert et al., 2015; Violle et al., 2012).

At species level, a leading approach to study ecological strategies is the competitor, stress‐tolerator, and ruderal (CSR) theory proposed by Grime (1974). In this theory, two main factors shape plant strategies: stress, namely the availability of accessible environmental resources, and disturbance, this being the periodic destruction of biomass due to biotic and abiotic causes (Grime, 1977). Accordingly, a plant species can be classified into one of three major groups: stress‐tolerators (conservative use of resources, slow growth, and high survival in resource‐poor and less‐disturbed environments); competitors (intermediate use of resources, highly efficient in using resources and allocating them to vegetative growth under conditions of low stress and little disturbance); and ruderals (acquisitive use of resources, rapid growth, and relatively short‐lived, being specialists of highly disturbed habitats) (Grime & Pierce, 2012). To quantify CSR strategies, a viable methodology based on leaf economic spectrum and size‐related traits has been developed (Pierce et al., 2013), and this system has proven useful to represent and compare ecological strategies across different ecological scales, from species (Pierce et al., 2014) to habitats (Negreiros et al., 2014), successional stages (Wen et al., 2022), and biomes (Pierce et al., 2016). However, it is still unclear whether this simple method designed for allocating C‐S‐R strategies at the species level is also effective when applied within individual species.

The CSR strategy has been widely applied to explore how plants respond to differing environmental conditions (Alessandro et al., 2022; Caccianiga et al., 2006; Rosenfield et al., 2019). For example, the dominant species in a resource‐limited system shifts from those with a high stress‐tolerant strategy to those with C and R strategies as the availability of water and soil nutrients increases (Butterfield & Briggs, 2011; Rosado et al., 2017). Similarly, warmer temperatures lead to reduced R and S components while augmenting plant competitiveness (Rosenfield et al., 2019; Zhang & Wang, 2021). Both May et al. (2017) and Vasseur et al. (2018) found that pronounced variation in strategies among Arabidopsis accessions was evident along the S‐R axis, but they arrived at opposite conclusions about relationship between strategy and climate. In sum, much research has shown that a greater stress‐tolerant ability characterizes plant species or populations in relatively harsh environments, whereas acquiring more environmental resources via competition is a crucial ability in stable and productive environments. However, since these findings have focused exclusively on adults in a population, the possible divergence in ecological strategies among all individuals remains understudied.

In fact, the variation in plant ecological strategies along environmental gradients may vary according to individual ontogenetic stage. The susceptibility of tree seedlings to injury and stress is widely recognized across different vegetation types and environmental gradients due to their small and fragile organs (Coelho et al., 2008; Moles & Westoby, 2004; Stearns, 1976). Consequently, juveniles are at a higher risk of mortality when encountering environmental disruptions compared to their adults who can better cope with variability in the availability of accessible nutrients and water in their environment as they have accumulated sufficient biomass and come to occupy a stable ecological niche (Kenzo et al., 2015; McDowell et al., 2002; Niklas & Enquist, 2002; Pardos et al., 2009). Accordingly, changes in the structure and functional traits of trees could develop at various stages throughout their ontogeny (Damián et al., 2017; Martin & Thomas, 2013; Meinzer et al., 2011). For example, the decline in SLA (specific leaf area) and photosynthetic rate from juvenile to adulthood is pronounced in tree species (Wei et al., 2020). Several studies have also found an acquisitive‐conservative strategy spectrum across tree ontogeny (He & Yan, 2018; Liu et al., 2020; Mason et al., 2013). We may therefore predict that juveniles of a tree species will be inclined towards R given their innate vulnerability irrespective of local environmental conditions, whereas their adults are likely to diverge into differing strategies in response to long‐term environmental filtering.

The establishment and development of pioneer tree species and their role in improving harsh environments is paramount for the ecological restoration of degraded forests (Lu et al., 2017; Xu et al., 2015). Pioneers, being very sensitive to environmental changes, will in turn be influenced by continual flux in abiotic factors as restoration proceeds, namely increasing soil fertility and decreasing light availability (Espírito‐Santo et al., 2013; Pardos et al., 2009). To optimize their own fitness, individuals at different ontogenetic stages within a pioneer population could adopt contrasting strategies in response to environmental change during the course of restoration (Letcher et al., 2015; Lopez‐Martinez et al., 2013; Wang et al., 2021, 2023). In this way, by studying tree trait dynamics under ecological restoration, we can investigate the transition of strategies within a pioneer species along an environmental gradient. Such variation in strategies among individuals will determine the fitness and population growth of pioneer species and is essential for the later succession of non‐pioneer species (Smith & Nichols, 2005; Wang et al., 2021; Winkler et al., 2015).

Here, we aimed to investigate intraspecific variation in the ecological strategies of *Pinus massoniana*, a pioneer conifer tree species used for ecological restoration in Changting County, Fujian Province, China. To represent distinct stages of that ecological restoration process, we selected three plots with different stand structures and habitat conditions. The CSR system was built using six traits: SLA, leaf dry matter content (LDMC), leaf nitrogen concentration (LNC), leaf phosphorus concentration (LPC), leaf area (LA), and canopy height, along with the individuals' calculated C, S, and R scores in each ontogenetic stage in the three restoration stages (plots). We used that to test hypotheses, the pattern of variation in ecological strategies of *P. massoniana* along the restoration gradient differs among ontogenetic stages: adults transition from being more stress‐tolerant (S) to more competitive (C), while juveniles tend to be more Ruderal (R) and maintain this strategy as restoration progresses.

## 2 MATERIALS AND METHODS

### 2.1 Study area and species

**FIGURE 1:**
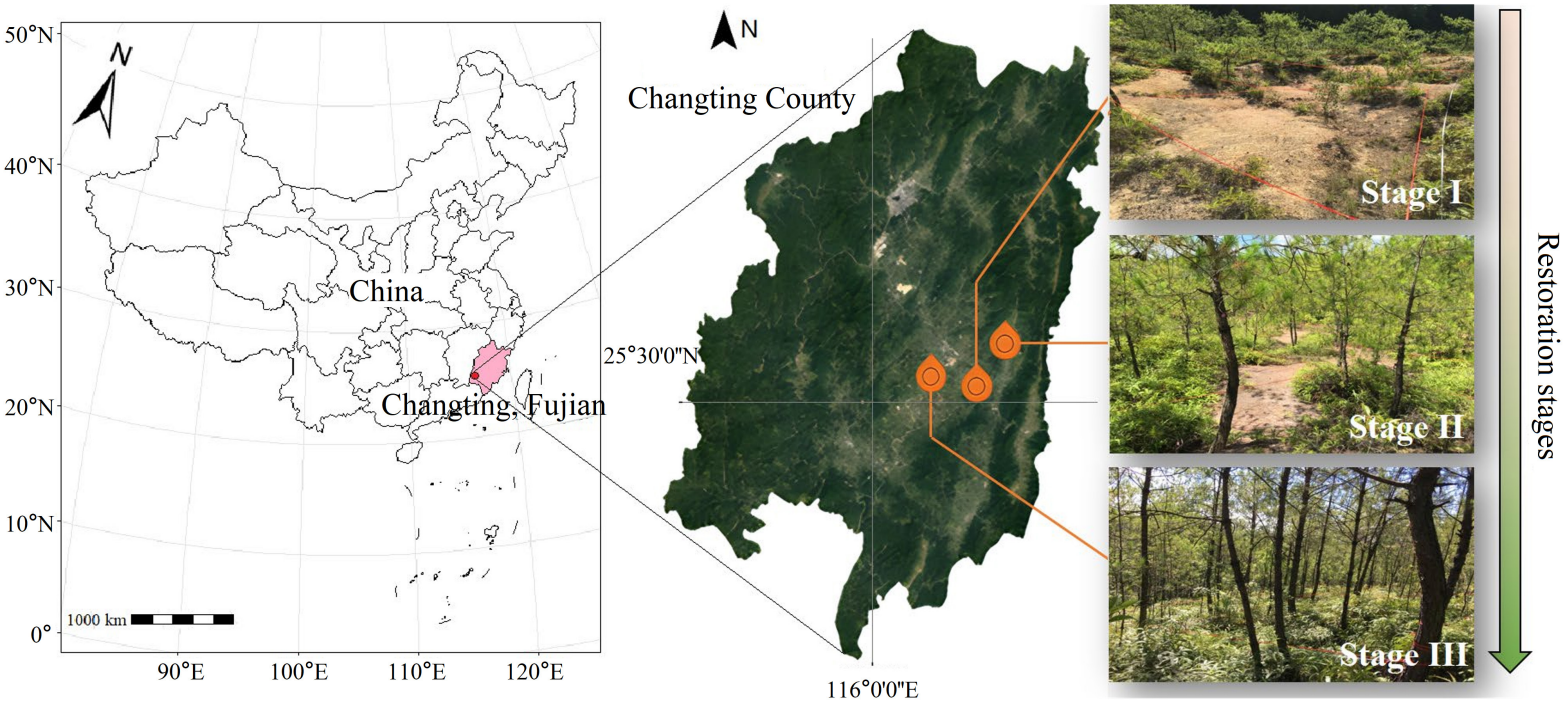
Geographical location of the study area in Fujian Province, China, and the plot locations sampled for Pinus massoniana trees. Plot details can be found in Table 1.

Our study was conducted in three subtropical secondary forest plots in Changting County (25°18′40″–26°02′05″ N, 116°00′45″–116°39′20″ E), in the western part of Fujian Province, China (Figure 1). This study area has a subtropical marine monsoon climate, with an average annual temperature of 18.3°C and precipitation of 1685.6 mm, the latter occurring mostly in April–June; hence, rainfall and heat are not synchronized, which can worsen the drought stress incurred by plants during the growing season. Zonal soils consist of red soils (according to the Chinese Soil Taxonomy [CST] system), which roughly correspond to oxisols, ultisols, and certain alfisols (as recognized by the USDA Soil Taxonomy system), derived from the weathered crust of coarse granite (Shi et al., 2006). Historically, the area has incurred severe soil erosion and vegetation degradation due to anthropogenic activities (Vajpeyi, 2001). Fortunately, after 40 years of afforestation, the local vegetation has undergone natural succession and *P. massoniana* is now the dominant tree species (Ma & Xuan, 2008).

*Pinus massoniana* is a strongly shade‐intolerant species, monoecious, flowering in April–May each year, with cones that usually develop over 2 years from which young seedlings are usually established in March of each year. Coupled with its advantages of establishing barren land, drought tolerance, and a rapid growth rate, *P. massoniana* is becoming the pioneer species of choice for local ecological restoration projects (Atsushi et al., 2005; Wang et al., 2023). During ecological restoration, soil fertility, understory vegetation cover and height, species abundance, and the number of broadleaf species in this native forest type all gradually increase. Based on these indicators, our plots (restoration stage I–III) represent distinct restoration stages of the early ecological restoration process in Space‐For‐Time (SFT) substitution, respectively, to simulate the sequence of ecological restoration (Table 1). These three plots were set up in July 2021 and their sizes are restoration stage I (900 m^2^), restoration stage II (800 m^2^), restoration stage III (900 m^2^) respectively in order to avoid excessive habitat heterogeneity within the sample plots.

**TABLE 1:** The conditions of the three sampling plots spanning the ecological restoration gradient.

Restoration stage	Plot size (m^2^)	Soil organic matter (g/kg)	Soil available nitrogen (mg/kg)	Soil available potassium (mg/kg)	Water content of soil (%)	Density of *Pinus massoniana* ^a^ (/ha)	Density of *P. massoniana* ^b^ (/ha)	Understory vegetation cover^c^ (%)	Max bd in *P. massoniana* (mm)	Number of broadleaf tree species	Simpson index (%)
I	900	4.26	5.08	1.56	7.79	800	111	52	159.74	0	70
II	800	6.41	11.38	1.59	8.11	2325	175	57	159.51	1	72
III	900	6.59	15.75	3.00	10.42	3025	625	91	188.73	7	77

^a^ Calculated for those individuals with a basal diameter >1 cm.

^b^ Calculated for those individuals with a basal diameter >10 cm.

^c^ Herbaceous plant coverage.

We used individual size (basal diameter, BD) to represent different ontogenetic stages of *P. massoniana* (Dayrell et al., 2018; Zhang & Wang, 2021). The BD ranges were similar across the three plots: 11.15–159.74, 12.41–159.51, and 10.26–165.24 mm. Four distinct ontogenetic stages were distinguishable: *juveniles* (10 < BD < 30 mm) were individuals with a high mortality risk; *saplings* (30 < BD < 60 mm) were those with a relatively higher survival prospects (rarely 100%); *adult‐1 trees* (60 < BD < 100 mm) were those that had just entered the reproductive stage; *adult‐2 trees* (BD > 100 mm), these being reproductively mature for many years (Zhao et al., 2021).

### 2.2 Traits' selection and measurement

According to Pierce et al. (2013), when constructing the CSR system, it is important to select traits that are related to leaf economic spectrum and individual size. Many studies have demonstrated the existence of a conservative‐acquisitive leaf economic spectrum in coniferous trees along environmental gradients and during individual ontogeny (He et al., 2020; Kuusk et al., 2018; Liu et al., 2020; Qin & Shangguan, 2019). SLA is positively related to a more acquisitive resource‐use strategy, a higher relative growth rate and leaf net photosynthetic rate (Liu et al., 2020). LDMC reflects a more conservative resource‐use strategy and is usually negatively correlated with relative growth rate (Qin & Shangguan, 2019). LNC is closely tied to the mass‐based maximum photosynthetic rate (Kuusk et al., 2018). Additionally, LNC and LPC of an individual plant reflect the N and P availability in its environment (He et al., 2020).

LA indeed constitutes a widely available indicator of the plant/organ size spectrum at the interspecific level (Díaz et al., 2016). However, to our knowledge, there is limited research on the variation of needle size, making it difficult for us to determine whether needle size effectively characterizes individual size of *P. massoniana* trees and their competitive ability. Based on this, we decided to include canopy height as a trait in our study which has been proven to be an effective competitive strategy for pioneer trees (even seedlings) when they faced reduced light availability (Agyeman et al., 1999; Carlyle & Fraser, 2006; Wu et al., 2020), and it tends to increase with individual ontogeny (Liu & Zeng, 2017). Finally, we selected six traits for our study: SLA, LDMC, LNC, LPC, LA, and canopy height.

All traits were measured following Pérez‐Harguindeguy et al. (2013) in August 2021. Canopy height (cm) is the shortest distance between the top of the upper leaves and the ground level. We sampled the *P. massoniana* trees and measured their leaf traits when leaves were fully expanded and mature. From each individual tree, 3 or 4 twigs were cut off and refrigerated in a 4°C portable incubator. All leaves of each individual tree were removed from above twigs and 20 leaves similar in size were randomly selected, and scanned by an Epson scanner. LA (cm^2^) was calculated using Image J (National Institutes of Health; https://imagej.net/software/fiji/downloads), and finally, we obtained an average of the 20 leaves. We used an analytical balance (0.1 mg) to measure the leaf fresh weight (measured immediately after removing the blade), saturated fresh mass (measured after 8 h of soaking), and dry mass (measured after oven‐drying to a constant weight) of the 20 leaves and again the mean values were derived. The SLA (cm^2^/g) was calculated as one‐sided fresh LA divided by its dry mass. The LDMC (dry mass per unit water‐saturated fresh mass; %) was calculated as dry mass by its saturated fresh mass. For each sample, its dried leaves were then ground into powder, for which the LNC (mg/g) was measured using an elemental analyzer (Vario EL III; Elementar, Hanau, Germany) and LP (mg/g) by a continuous flow analyzer (AutoAnalyzer 3, Germany).

### 2.3 Statistical analysis

All data analyses were carried out in R v4.2.3 (R Core Team, 2023). To assess the intraspecific variability of each functional trait, employing a one‐way analysis of variance (ANOVA), we conducted a comparison of LA, SLA, LDMC, LNC, LPC, and canopy height among restoration stages for each ontogenetic stage. Likewise, we also evaluated the variation of these traits among ontogenetic stages within each restoration stage. In both instances, pairwise comparisons of the means were performed using the Tukey HSD test.

To test whether there is a leaf economic spectrum and a size‐related axis, we first used a principal component analysis (PCA) to quantify linear relationships between all measured functional traits, using the ‘rda’ function in the ‘vegan’ package (Oksanen et al., 2019).

We then calculated the C, S, and R scores of every individual. To adapt the method to our specific research object, certain modifications were made to the original method described by Pierce et al. (2013). Specifically, we used canopy height rather than LA to characterize individual size and its competitive ability. The specific steps are as follows: (a) perform a PCA on the key traits (i.e., SLA, LDMC, LNC, LCC, LA, and canopy height), then extract those traits that contribute most to the first and second principal components, respectively; here, SLA, LDMC, and canopy height were extracted; (b) regress LDMC against the PCA axis 1, SLA against the PCA axis 1, and canopy height against the PCA axis 2, thereby yielding three regression equations with the highest *R* ^2^‐values (Figures S3–S5); and (c) use these regression equations to create a Microsoft Excel spreadsheet. In this spreadsheet, the regression equations are applied to produce ternary coordinates representing trade‐offs between the three main competing functional traits and thus three competing ecological functions (Pierce et al., 2013).

Finally, two‐way analysis of variance (ANOVA) was used to test the effects on C‐, S‐ and R‐scores due to ontogeny and restoration stages and the interaction of these two explanatory factors. However, we did not find a significant interaction effect on any CSR strategy of *P. massoniana* trees (Table S2). Therefore, one‐way ANOVA was inset used to examine restoration's effect, and likewise ontogeny's, with their stage means compared on a pairwise basis using the Tukey HSD test, again.

## 3 RESULTS

### 3.1 Variation of functional traits through restoration stages and ontogeny

For all ontogenetic stages, there was a consistent trend of increasing canopy height, SLA, LNC, and LPC, but decreasing LA and LDMC during the restoration process. The difference between restoration stage I and restoration stage III was almost significant (Table 2, Figure S1). At the same time, canopy height, LA, and LDMC increased while SLA, LNC, and LPC decreased from juvenile to adult‐2 within each restoration stage. Except for the LPC in restoration stage III, the variation of all other traits with individual ontogeny within each restoration stage was significant (Table 2, Figure S2).

**TABLE 2:** Mean values and standard deviation of six functional traits for each ontogenetic stage in each restoration stage for *Pinus massoniana*.

	SLA (cm^2^/g)		LDMC (%)		Canopy height (cm)		LA (cm^2^)		LPC (mg/g)		LNC (mg/g)	
	Mean	SD	Mean	SD	Mean	SD	Mean	SD	Mean	SD	Mean	SD
I J	51.155^a,c^	8.602	36.452^b,a^	2.452	178.588^a,a^	81.238	0.649^b,a^	0.119	0.808^a,bc^	0.243	8.897^a,b^	1.411
I S	43.258^a,bc^	5.522	38.162^b,ab^	1.793	302.864^a,b^	60.170	0.713^b,a^	0.152	0.610^a,b^	0.146	8.630^a,a^	1.048
I A‐1	39.583^a,b^	5.440	39.629^b,bc^	2.141	455.400^a,c^	62.892	0.801^b,ab^	0.182	0.666^a,b^	0.153	8.937^ab,ab^	1.013
I A‐2	34.850^a,a^	4.797	41.247^b,c^	2.682	511.444^a,c^	64.185	0.987^b,c^	0.179	0.497^a,a^	0.083	7.553^a,a^	1.169
II J	57.734^a,d^	7.315	33.218^a,a^	3.608	168.944^a,a^	59.428	0.560^b,a^	0.115	0.807^a,bc^	0.134	8.814^a,c^	0.889
II S	51.315^b,bc^	7.127	34.993^a,ab^	2.324	334.960^a,b^	82.383	0.631^ab,a^	0.149	0.794^b,b^	0.091	8.362^a,bc^	0.985
II A‐1	48.454^b,b^	5.629	36.739^a,bc^	2.174	449.909^a,c^	80.056	0.800^b,b^	0.185	0.720^a,a^	0.067	8.041^a,ab^	0.724
II A‐2	40.761^ab,a^	5.999	38.383^a,c^	2.684	576.143^a,d^	82.051	0.879^ab,bc^	0.184	0.700^b,a^	0.072	7.625^a,a^	1.028
III J	68.653^b,d^	9.522	33.132^a,a^	2.229	264.600^b,a^	85.653	0.447^a,a^	0.132	0.957^b,a^	0.162	10.390^b,ab^	1.878
III S	60.128^c,c^	7.594	34.154^a,ab^	1.813	451.528^b,b^	95.989	0.555^a,ab^	0.161	0.884^c,a^	0.107	10.482^b,b^	1.583
III A‐1	51.927^b,b^	9.012	35.454^a,b^	1.810	568.818^b,c^	76.740	0.650^a,b^	0.153	0.889^b,a^	0.092	9.911^b,ab^	1.288
III A‐2	43.635^b,a^	8.342	37.962^a,c^	2.083	695.5^b,d^	57.465	0.810^a,c^	0.178	0.853^c,a^	0.088	9.094^b,a^	1.406

*Note*: The letters following the mean values indicate the results of Tukey HSD test conducted on the mean values of all traits. Different letters indicate significant differences among trait values. The former represents the results of comparisons among different restoration stages for each ontogenetic stage, while the latter represents the results of comparisons among different ontogenetic stages within each restoration stage.

### 3.2 Linear relationships between all functional traits

**FIGURE 2:**
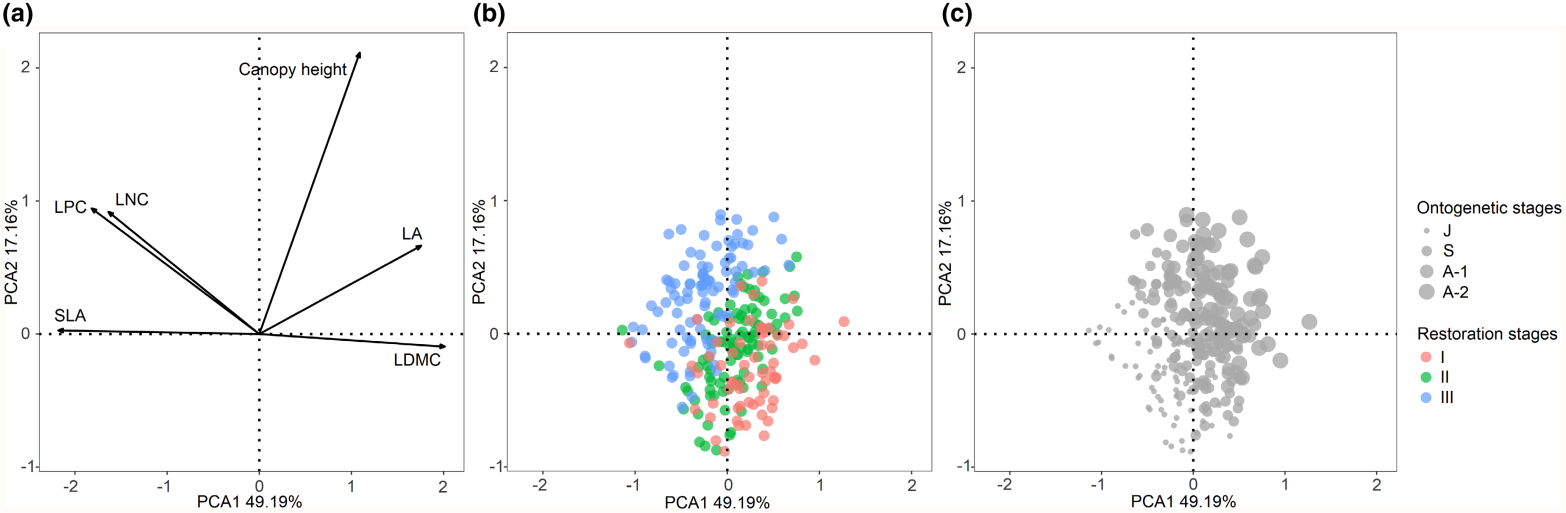
Principal component analysis (PCA) of trait variation for 252 *Pinus massoniana* individuals. (a) The relationship between all measured functional traits. LA, leaf area; LDMC, leaf dry matter content; LNC, leaf nitrogen concentration; LPC, leaf phosphorus concentration; SLA, specific leaf area; and canopy height. (b) Locations of *P. massoniana* individuals in each restoration stage in trait‐multivariate space. Red, green and blue dots represent individuals in restoration stage I, II and III respectively. (c) Locations of *P. massoniana* individuals at each ontogenetic stage in trait‐multivariate space. Dots of different sizes symbolize individuals at distinct ontogenetic stages of *P. massoniana*, ranging from the smallest to the largest: juveniles, saplings, adult‐1 trees, and adult‐2 trees.

The first two principal components accounted for 66.35% of the total variation in the six traits. PCA1 was a leaf economic axis: LDMC was positively associated with PCA1, whereas SLA, LNC, and LPC were negatively associated with it. Surprisingly, LA was also strongly and positively associated with PCA1. PCA2 represents the individual size of *P. massoniana* trees, which was closely and positively correlated with the canopy height (Figure 2a, Table S1). The PCA of functional traits showed a clear separation between restoration stage I, II, and III. When compared with restoration stage I and II, juveniles in restoration stage III featured more acquisitive leaf economics, higher canopy height, and smaller leaf area (Figure 2b). The same pattern of trait variation was also observed for other three ontogenetic stages. At each restoration stage, in going from juvenile to adult, leaf traits tended to become more economically conservative and larger in size along with a greater canopy height (Figure 2c).

### 3.3 Variation in CSR components through restoration stages and ontogeny

**FIGURE 3:**
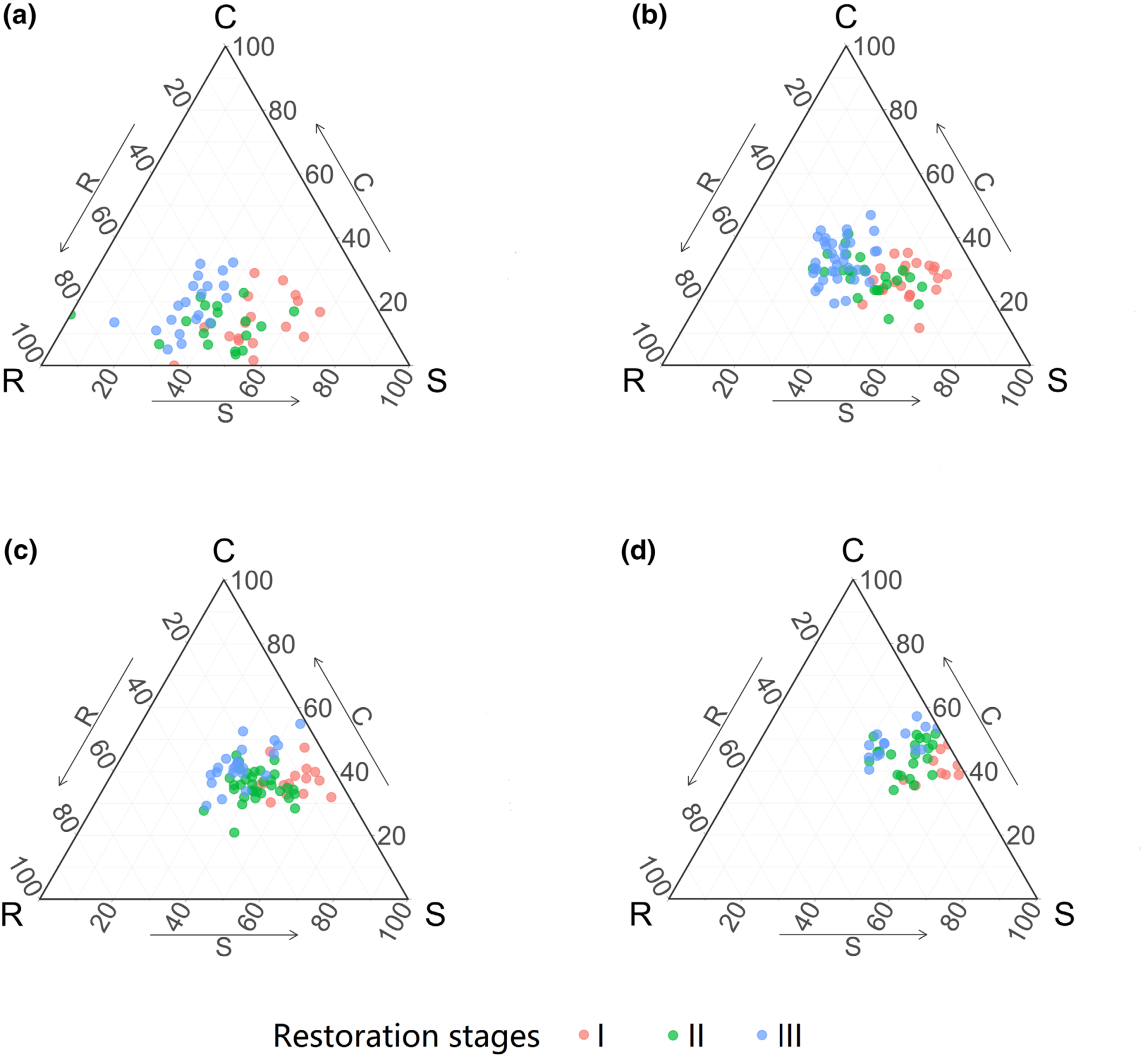
Ternary plots showing the variation in CSR scores for *Pinus massoniana* individuals at different ontogenetic stages across the three restoration stages, and (a) for juveniles, (b) for saplings, (c) for adults‐1, and (d) for adults‐2.

All individuals from three restoration stages fell into the SR, CSR, and SC regions of the triangle (Figure 3a–d). Juveniles were mainly concentrated around the SR region and their mean C: S: R strategy values were 14%: 52%: 34%, 13%: 41%: 46%, and 19%: 32%: 49%, whereas adults‐2 were positioned mainly in the SC region, having 41: 53: 6%; 44: 43: 13%, 49: 37: 14% values for each consecutive restoration stage, respectively (Figure 3a,d, Table 3).

**TABLE 3:** Mean values and standard deviation of C, S, and R scores for each ontogenetic stage in each restoration stage for *Pinus massoniana*.

	S		C		R	
	Mean	SD	Mean	SD	Mean	SD
I J	51.857	8.889	13.657	8.214	34.486	12.621
I S	53.448	6.377	26.609	5.548	19.943	7.172
I A‐1	50.254	6.214	37.332	4.845	12.414	6.417
I A‐2	52.547	4.493	41.151	4.358	6.302	6.255
II J	40.994	13.362	12.749	5.978	46.255	12.985
II S	41.748	9.810	28.140	5.854	30.112	6.743
II A‐1	41.116	6.356	35.445	4.882	23.439	6.335
II A‐2	42.687	5.825	44.487	5.453	12.826	6.625
III J	31.428	5.878	19.157	8.181	49.416	10.551
III S	31.716	5.563	32.944	6.627	35.340	6.686
III A‐1	33.701	5.017	41.794	6.302	24.505	9.108
III A‐2	37.060	5.392	48.714	4.498	14.226	8.181

**FIGURE 4:**
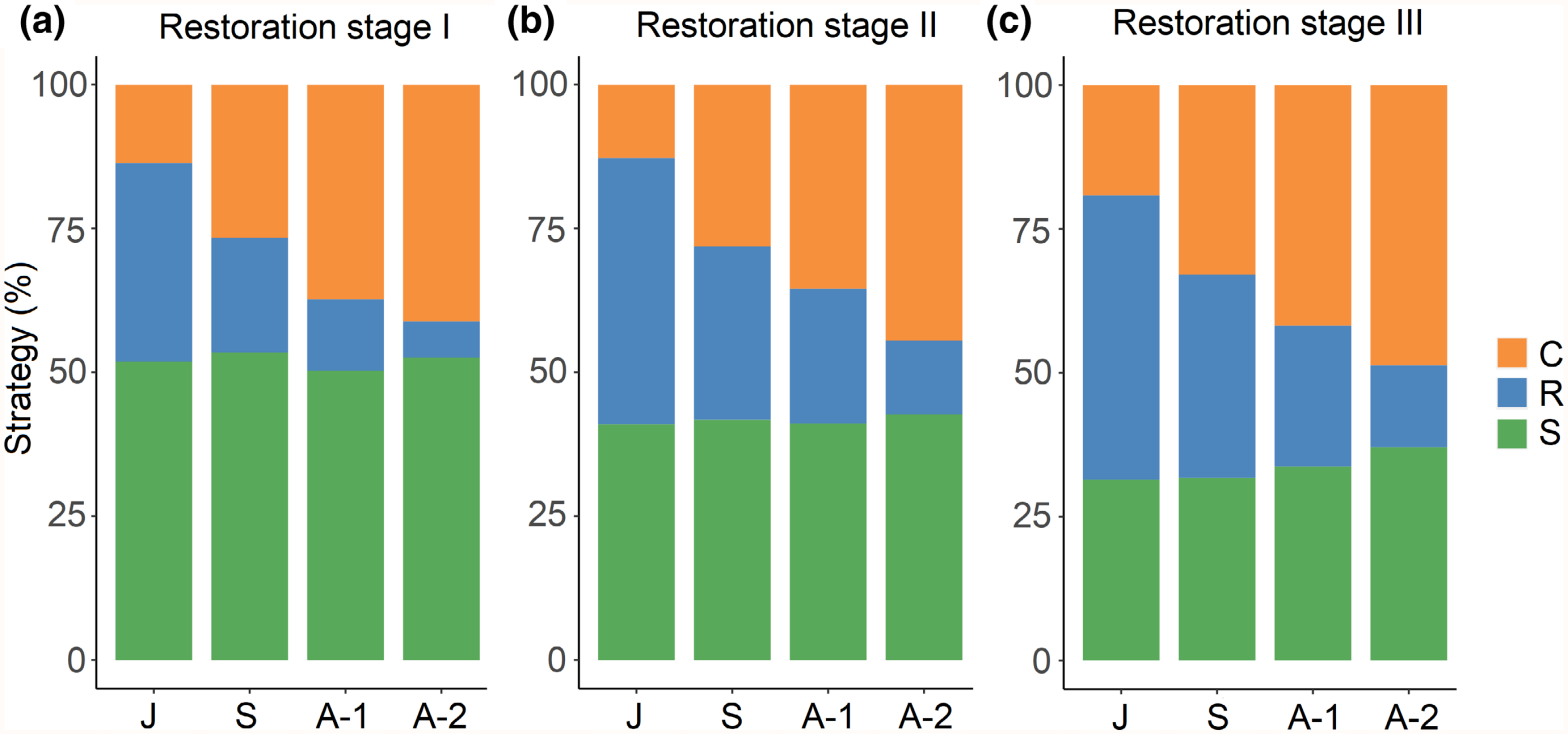
Means of the CSR strategy of *Pinus massoniana* individuals at four ontogenetic stages (juvenile, sapling, adult‐1, adult‐2) in each restoration stage. Green, blue, and orange bars indicate the proportion of S, R, and C components contributing to the overall ecological strategy of *P. massoniana* individuals, respectively.

There was significant variation in the S score for juveniles across restoration stages, with the S score decreasing as restoration progressed (Figure S6a). The C score of juveniles showed a significant variation between restoration stage I and III, as well as between restoration stage II and III. Only between restoration stage I and III, there was a significant variation in R score for juveniles (Figure S6e,i). The variation in S, C, and R components for other ontogenetic stages followed the same changing pattern (Figure S6b–d,f–h,j–l). In every restoration stage, juvenile‐to‐adult transitions were characterized mainly by decreased R scores but increased C scores (Figure S7d–i). Ontogenetic variation in ecological strategies was not significant (*p* < .05) for S score in restoration stage I or II, while a significant yet small difference was found between juveniles and adults‐2 only in restoration stage III (Figure S7a–c). Additionally, the standard deviation (SD) of C, S, and R scores of individuals decreased through ontogeny (Table 3). In terms of the overall CSR strategy, the S score made the largest contribution to it for every ontogenetic stage in restoration stage I (Figure 4a), but in restoration stages II and III, the R score contributed the most for juveniles and the C score contributed the most for adults (Figure 4b,c).

## 4 DISCUSSION

Our results showed that the CSR strategy of *P. massoniana* transitioned distinctly along ecological restoration gradient for all four ontogenetic stages (Figure 3). Specifically, S component decreased but both C and R components increased (Table 3, Figure S6), suggesting a reduced stress‐tolerant ability but enhanced resource‐acquisitive ability and competitive advantage. Furthermore, the ecological strategy of adult trees transitioned from stress‐tolerance‐dominated to competition‐dominated, while that of juvenile trees shifted from stress‐tolerance‐dominated to ruderal‐dominated (Figure 4).

### 4.1 Shifts in the ecological strategy of adult *P. massoniana* along the restoration gradient

Most previous studies have concluded that adult trees have developed relatively stable strategies that are well‐adapted to their environments because of a long‐term environment they have experienced since the juvenile stage (Negreiros et al., 2014; Onate & Munne‐Bosch, 2009; Pierce et al., 2016). Our study supports this prevailing view because we found a less variability in ecological strategies of *P. massoniana* adult trees, as evidenced by a much smaller SD for their CSR strategy vis‐à‐vis their conspecific juveniles within every restoration stage (Table 3), indicating the convergent adaption of *P. massoniana* adults to habitats they inhabit.

At restoration stage I, the significant stress‐tolerant character (largest contribution of the C score) in strategy of adult *P. massoniana* implies their adaption to this unfavorable environment (Siefert, 2012). Arid microclimate, intense solar radiation, and barren soil forced them to store limited water and nutrients in order to withstand long‐term environmental stress (Faccion et al., 2021). Hence, they invest these scarce resources in long‐lived structures, leading to a higher leaf dry matter content (LDMC), and are then associated with conservative resource‐use strategy (Dayrell et al., 2018; Wright et al., 2005) whereby their growth rate is slowed to maximize the probability of survival in such a poor habitat (Christopher et al., 2014; Maynard et al., 2021). As ecological restoration proceeds, the stress‐tolerant character of *P. massoniana* adults gradually weakens because of enlarging pool of available environmental resources. Until restoration stage III, adults displayed a more competitive character (largest contribution of the C score). For pioneer species growing in a rapidly developing vegetation, realization of a high growth rate might be of prime importance (Carlyle & Fraser, 2006). A stable and productive environment in this stage may promote quick and continuous growth of *P. massoniana* trees, especially their height growth (Grime, 1977; Pierce et al., 2013; Zhao et al., 2021). In so doing, they can gain a competitive advantage in a gradually denser and darker forest (Zhao et al., 2021). However, the pressure of maintaining the functioning of such a large organism and reproductive activities in adulthood also makes them adopt relatively intermediate resource‐use strategies (Albert et al., 2010; Grime, 1977; Thomas, 2011).

### 4.2 Shifts in the ecological strategy of *P. massoniana* at early ontogenetic stages along the restoration gradient

According to the CSR theory, the observed increase in selection towards ruderal (R) in juveniles relative to adults indicates a greater allocation of resources to a faster growth rate during the early stage of ontogeny as a habitat‐independent strategy to cope with higher levels of disturbance (Bond, 2000; Grime, 1977; Lambers & Poorter, 1992; Violle et al., 2012). However, our results demonstrated a significant shift in the overall ecological strategy of juveniles as the restoration progressed (Figure 4, Figures S6 and S7), despite juveniles consistently displaying higher R scores than adults at each restoration stage (Table 3). These findings suggest that the ecological strategy of *P. massoniana* juveniles still be influenced by the specific stage of restoration in our study.

Contrary to our expectations, in the restoration stage I, the largest contribution of S component was also observed in strategy of juveniles and saplings. This unexpected finding may indicate that even individuals at early ontogenetic stages in such harsh conditions prioritize resistance to environmental stress rather than rapid resource acquisition and consumption (Faccion et al., 2021; Siefert, 2012). As ecological restoration proceeds, the ruderal character of juvenile trees is becoming more conspicuous. Up to restoration stage III, juvenile trees featured a pronounced ruderal character (largest contribution of the R score), which may be attributed to increases in soil moisture and nutrients, and more vegetation coverage. This favorable environment provided juveniles with sufficient available resources for investment in quick biomass accumulation (Rosenfield et al., 2019; Zhang & Wang, 2021), resulting in a higher SLA that indicates an acquisitive resource‐use strategy (Niinemets, 2010; Steppe et al., 2011; Wright et al., 2005). In this way, they can reach the next ontogenetic stage as soon as possible that is less susceptible to disturbances such as heavy rains, heatwaves, and typhoons in this study area (Dayrell et al., 2018; Pardos et al., 2009). Moreover, as a strongly light‐demanding species, the faster growth rate of *P. massoniana* juveniles may also be an attempt to break the surrounding understory vegetation, enabling them capture enough light resource in canopy gap (Carlyle & Fraser, 2006). The almost equal proportion of C, S, and R components in the ecological strategy of saplings in restoration stage III may indicate that they faced moderate pressure of environmental stress and disturbance and competition relative to the other individuals in the study (Pierce et al., 2013).

Summing up then, our study indicates that with the process of restoration, *P. massoniana* adults transitioned their ecological strategies from tolerating environmental stress to competing for light, while juveniles transitioned from tolerating environmental stress to accumulating biomass fast through acquisitive resource use. The heterogeneity in C, S, and R components found in this particular case study, albeit significant, does not affect the substantial contribution from the S component across all restoration stages and ontogenetic stages. The high proportions obtained for the S score (always >30%) in the overall CSR strategy reflect the unique tolerance of this species to barrenness and drought (Atsushi et al., 2005). Despite the recent mounting recognition of the importance of incorporating intraspecific trait variation in plant functional trait approaches, empirical research on the variation of intraspecific traits and ecological strategies along short environmental gradients remains limited. Our study shows that the leaf economic spectrum and size‐related traits can effectively be used to study the intraspecific variability of ecological strategies in a conifer tree, *P. massoniana*. While other studies on intraspecific differences in strategies have focused on genetic differences (May et al., 2017; Vasseur et al., 2018), our study shifts its focus towards the impact of the restoration process, helping us gain a deeper understanding of plant adaptations to environmental conditions at local scale or within a short environmental gradient.

Different from general inter‐species CSR classifications, we constructed an intraspecific CSR system specifically for *P. massoniana* along ecological restoration because we found that canopy height, rather than leaf area, is an important trait for characterizing individual size and thus competitive ability of *P. massoniana* in this context. Just as Vasseur et al. (2018) pointed out classification methods based on leaf traits can be a powerful means of screening large databases at global scale, but might be of limited value in the examination of subtle variations within species and/or in specific taxa. Therefore, it is important for researchers to carefully consider and analyze trait–trait relationships and their ecological significances before applying a CSR classification to individual species within a specific context. Additionally, the intraspecific CSR system here may not be directly applicable to other species or broader interspecific analyses, so we look forward to further supplementation and validation of relevant researches.

## 5 CONCLUSION

Our study reveals a pattern of intraspecific variation in strategies during the process of forest restoration. *P. massoniana* trees' strategy is quite plastic phenotypically, as it undergoes shifts in response to stages of ecological restoration. Specifically, adults shift from tolerating environmental stress to competing for light, while juveniles shift from tolerating environmental stress to accumulating biomass through rapidly acquisitive resource use. Our findings can help us to discern *P. massoniana*'s strategies for adapting to different environments and their underlying trait‐driven mechanisms. In addition, our study also highlights the importance of selecting appropriate traits based on research subjects and contexts when studying intraspecific variation in ecological strategies using the CSR framework.

## AUTHOR CONTRIBUTIONS

**Sihang Lu:** Data curation (lead); investigation (lead); visualization (lead); writing – original draft (lead). **Jiazheng Wang:** Methodology (equal). **Ao Liu:** Investigation (equal). **Feiya Lei:** Investigation (equal). **Rong Liu:** Visualization (equal); writing – review and editing (equal). **Shouzhong Li:** Conceptualization (equal); supervision (equal).

## CONFLICT OF INTEREST STATEMENT

The authors declare no conflict of interest.

## Supporting information


Appendix S1

Appendix S2

Appendix S3

Appendix S4

Appendix S5

## Data Availability

All data generated or analyzed during this study are included in [Link], [Link], [Link], [Link].
