# Supplementary material for: Intraspecific transitioning of ecological strategies in Pinus massoniana trees across restoration stages
Source: Ecol Evol. 2024 May 5;14(5):e11305. doi: 10.1002/ece3.11305 (PMC11070636; doi:10.1002/ece3.11305)
Supplement: Supplementary file 1 — Appendix S1 [file ECE3-14-e11305-s001.docx]

Table S1. Spearman’s correlation coefficients between traits and PCA 1 and PCA 2 of the principal components analysis (PCA).

Table S2. Results of two-way ANOVAs.

Figure S1. Boxplots showing the variation in six functional traits for *Pinus massoniana* individuals across three restoration stages for each ontogenetic stage.

Figure S2. Ontogenetic variation in six functional traits of *Pinus massoniana* individuals in each of the three restoration stages.

Figure S3. Regression between LDMC and PCA 1.

Figure S4. Regression between SLA and PCA 1.

Figure S5. Regression between Canopy height and PCA 2.

Figure S6. Boxplots showing the variation in the C-, S-, R- scores for *Pinus massoniana* individuals across three restoration stages for each ontogenetic stage.

Figure S7. Ontogenetic transitions in C-, S-, and R- scores of *Pinus massoniana* individuals in each of the three restoration stages.

**Table S1.** Spearman’s correlation coefficients between traits and PCA 1 and PCA 2 of the principal components analysis (PCA).

| Traits | PCA1 | PCA2 |
| --- | --- | --- |
| LA  SLA  LDMC  LNC  LPC  Canopy height | 0.69 ***  -0.86 ***  0.79 ***  -0.64 ***  -0.72 ***  0.43 *** | 0.26 ***  0.01 n.s.  -0.04 n.s.  0.36 ***  0.37 ***  0.83 *** |

**Denotes significance at the P ≤0.01 level, ***P ≤0.0001. Traits are leaf area (LA), specific leaf area (SLA), leaf dry matter concent (LDMC), leaf nitrogen concentration (LNC), leaf phosphours concentration(LNC), and Canopy height.

**Table S2.** Results of two-way ANOVAs: effects of restoration stages and ontogenetic stages on C-, S-, R- scores. “***” denotes P ≤ 0.001, “—” denotes P > 0.05 (i.e., not significant) for the F-ratio.

|  | S | C | R |
| --- | --- | --- | --- |
| Restoration stage | *** | *** | *** |
| Ontogenetic stage | — | *** | *** |
| Restoration stage × Ontogenetic stage | — | — | — |

**
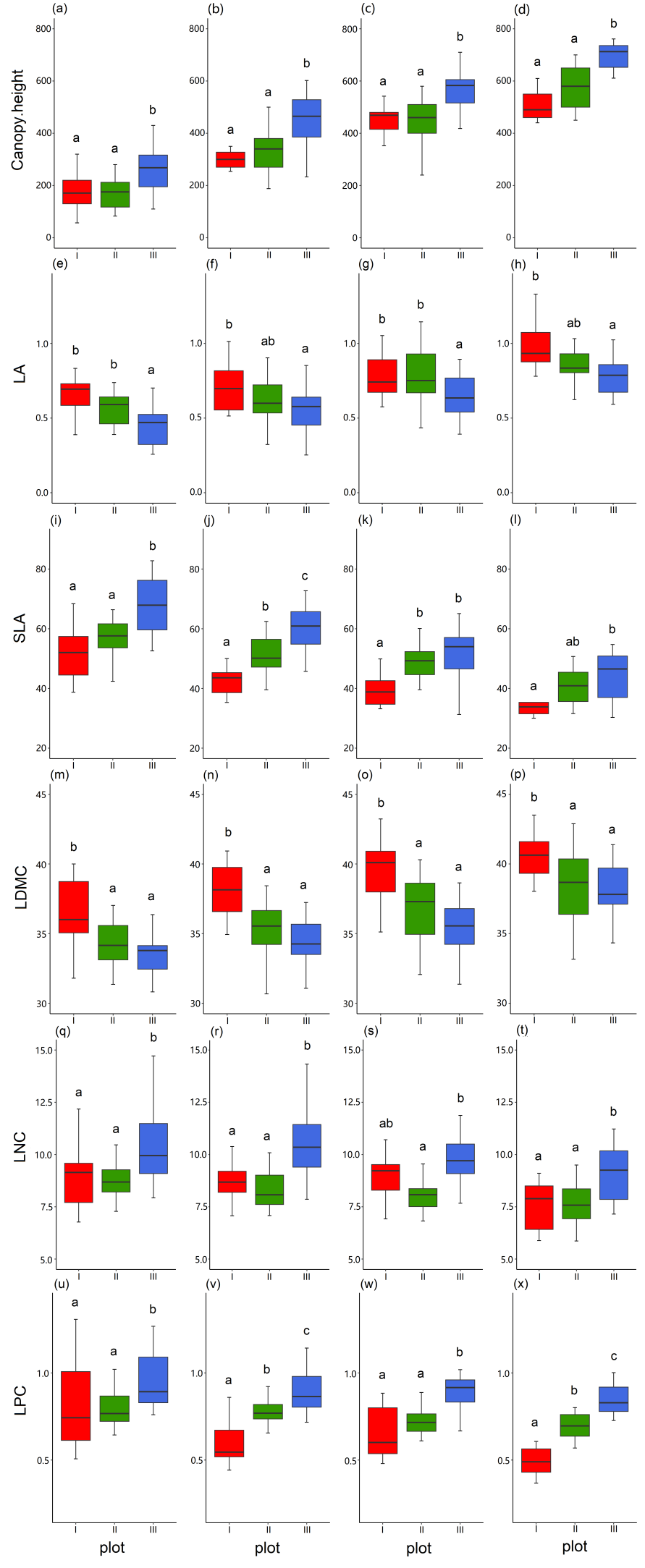
Figure S1** Boxplots showing the variation in six functional traits for Pinus massoniana individuals across three restoration stages for each ontogenetic stage: (a, e, i, m, q, u) juveniles; (b, f, j, n, r, v) saplings; (c, g, k, o, s, w) adults-1; (d, h, l, p, t, x) adults-2. Within each panel, different letters indicate significant differences among trait values of restoration stage I, II, III.

**
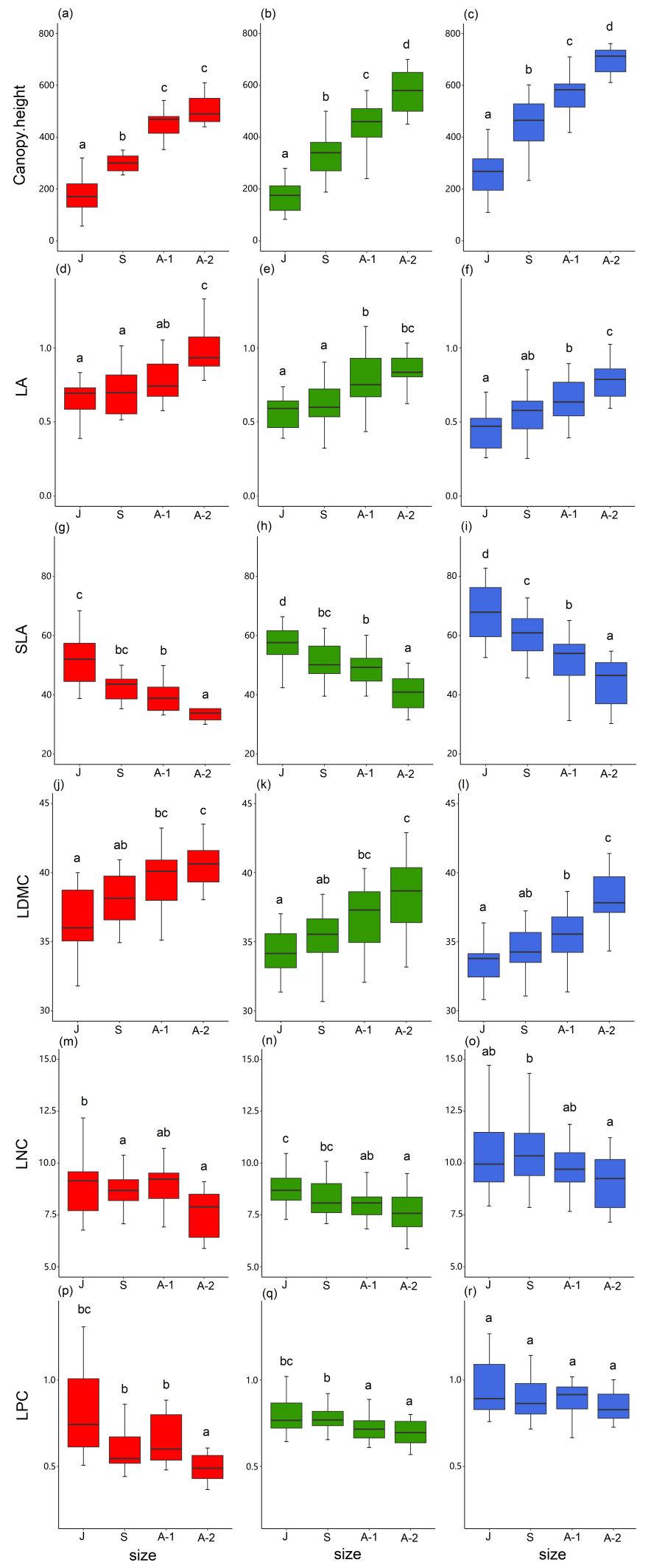
Figure S2**

Ontogenetic variation in six functional traits of Pinus massoniana individuals in each of the three restoration stages: (a, d, g, j, m, p) restoration stage I; (b, e, h, k, n, q) restoration stage II; (c, f, i, l, o, r) restoration stage III. Within each color panel, significant differences among four consecutive ontogenetic stages (juvenile, sapling, adult-1, adult-2) for each trait are indicated by different letters.


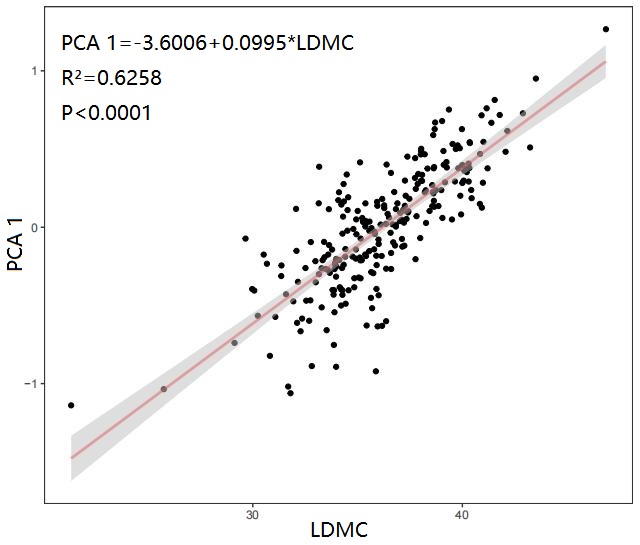


**Figure S3.** Regression between LDMC and PCA 1.


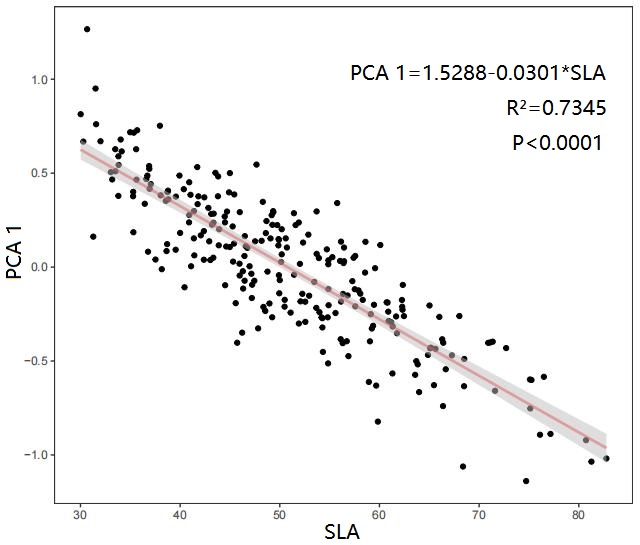


**Figure S4.** Regression between SLA and PCA 1.


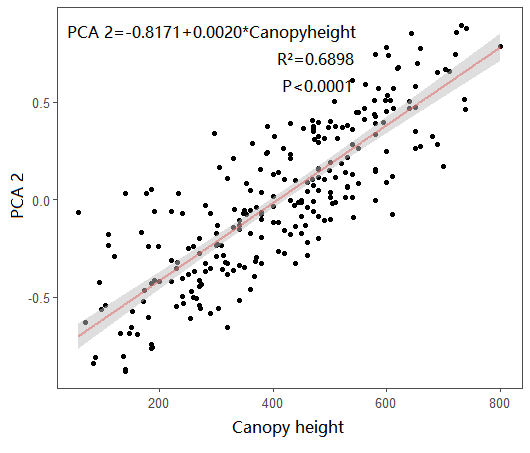


**Figure S5.** Regression between Canopy height and PCA 2.


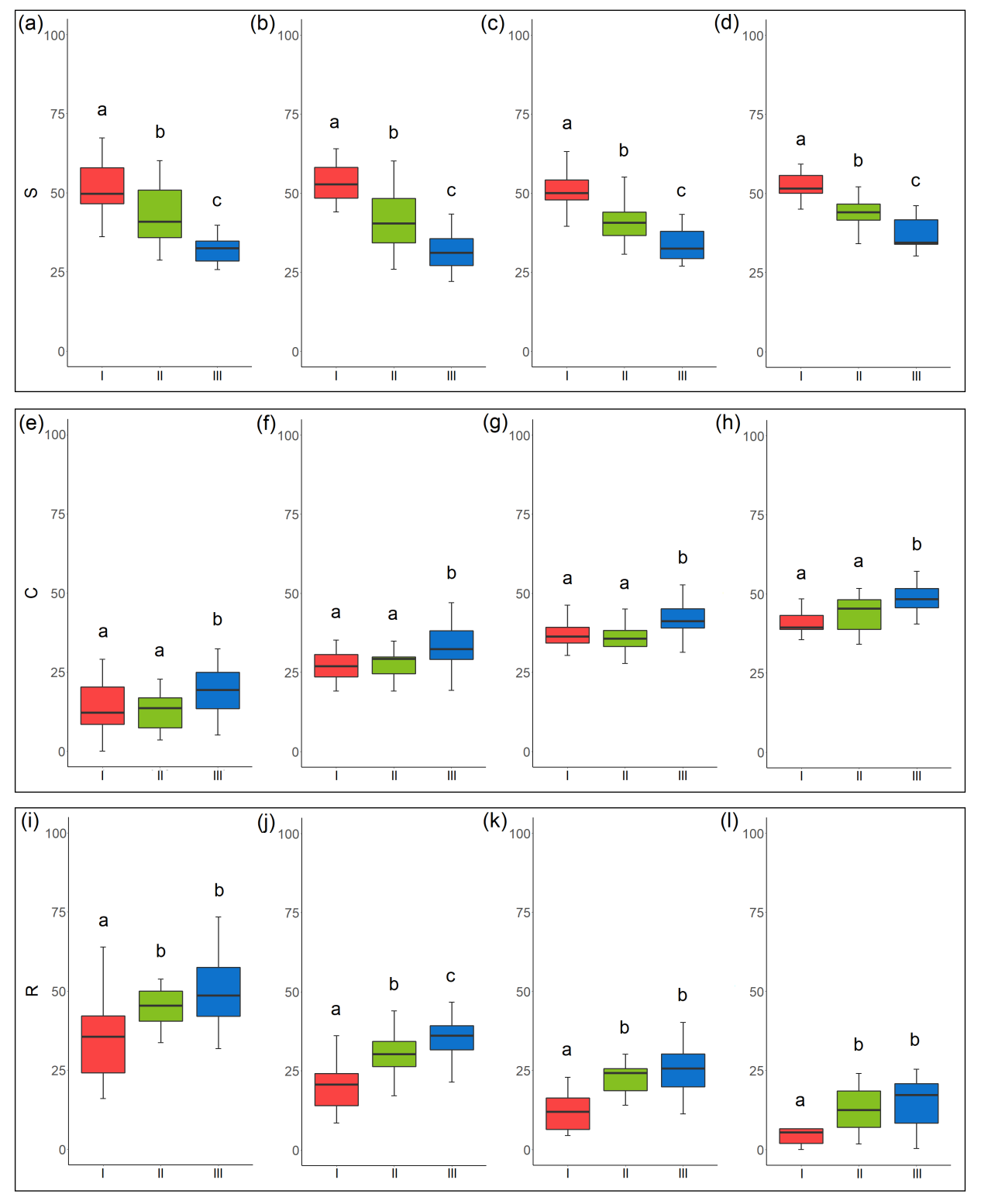


**Figure S6** Boxplots showing the variation in the C-, S-, R- scores for Pinus massoniana individuals across three restoration stages for each ontogenetic stage: (a, e, i) juveniles; (b, f, j) saplings; (c, g, k) adults-1; (d, h, l) adults-2. Within each panel, different letters indicate significant differences among scores of restoration stage I, II, III.


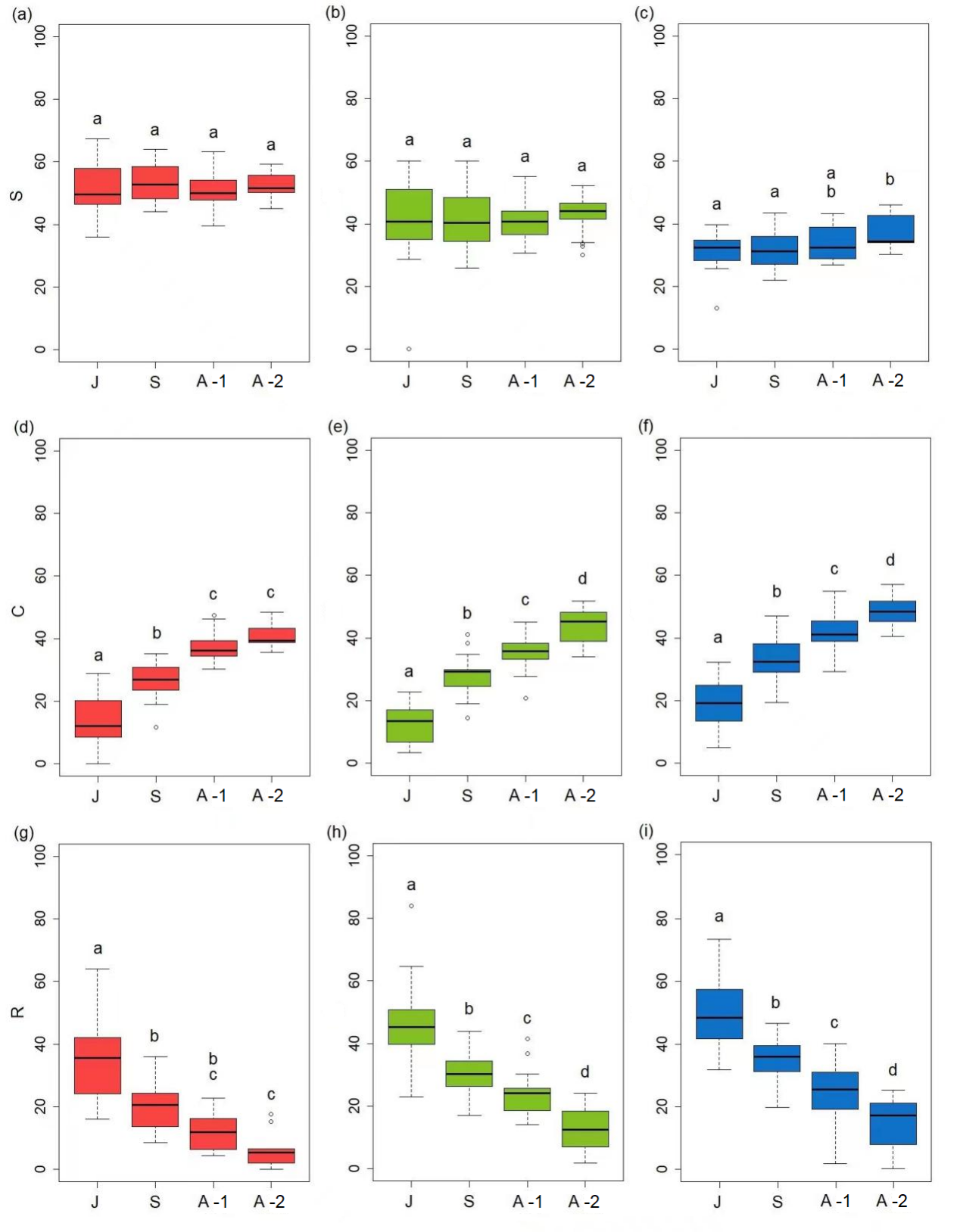


**Figure S7** Ontogenetic transitions in C-, S-, and R- scores of Pinus massoniana individuals in each of the three restoration stages: (a, d, g) restoration stage I; (b, e, h) restoration stage II; (c, f, i) restoration stage III. Within each color panel, significant differences (P < 0.05) among four consecutive ontogenetic stages (juvenile, sapling, adult-1, adult-2) for each CSR component are indicated by different letters.
