# Supplementary material for: Intraspecific transitioning of ecological strategies in Pinus massoniana trees across restoration stages
Source: Ecol Evol. 2024 May 5;14(5):e11305. doi: 10.1002/ece3.11305 (PMC11070636; doi:10.1002/ece3.11305)
Supplement: Supplementary file 4 — Appendix S4 [file ECE3-14-e11305-s005.docx]

**Details about spreedsheet calculating C, S and R scores**

Based on the outcome of the PCA the values of canopy height were regressed against PCA axis 2, LDMC was regressed against PCA 1 (a positive correlation), and SLA against PCA 1 (a negative correlation), in order to produce three regression equations. These represented curves of best fit (the highest values of R^2^).

The regression equations were then used to produce functions, in a Microsoft Excel worksheet, capable of predicting PCA 1 and 2 coordinates derived from values of canopy height, LDMC and SLA.

As PCA values may be negative or positive, the minimum (i.e. the most negative) values along PCA axes were then determined for each trait and these were used as a constant, added to all values of each trait in order to translate the canopy height, LDMC and SLA dimensions into an entirely positive space. The next spreadsheet function determined the maximum values, giving the range of values for each trait.

In order to produce ternary coordinates (i.e. three coordinates for a triangular graph), a function was then added to the spreadsheet that summed the three dimensions and divided by 100, allowing determination of the proportional contributions of canopy height, LDMC and SLA for each individual.

The resulting ternary coordinates represent a trade-off between three competing functional traits and thus three competing ecological functions.

We downloaded the original spreedsheet created by Pierce et al. (2013) (https://besjournals.onlinelibrary.wiley.com/doi/10.1111/1365-2435.12095, Appendix S3, CSR Triangulator VP spreadsheet (CSR classification tool)) and made changes to it. Specifically, we replaced values of three traits, traits-PCA axes regression equations, and the minimum and maximum values along PCA axes for each trait. To avoid changing the original spreedsheet's calculation order, we added one additional column of SLA data. This column will not impact the calculations in the spreedsheet.
